# Virtual reality training for upper extremity in subacute stroke (VIRTUES): study protocol for a randomized controlled multicenter trial

**DOI:** 10.1186/s12883-014-0186-z

**Published:** 2014-09-28

**Authors:** Iris Brunner, Jan S Skouen, Håkon Hofstad, Liv I Strand, Frank Becker, Anne-Marthe Sanders, Hanne Pallesen, Tove Kristensen, Marc Michielsen, Geert Verheyden

**Affiliations:** Department of Global Public Health and Primary Care, University of Bergen, Bergen, Norway; Department of Physical Medicine and Rehabilitation, Haukeland University Hospital, Bergen, Norway; Sunnaas Rehabilitation Hospital, Nesoddtangen, Norway; Institute of Clinical Medicine, University of Oslo, Oslo, Norway; Hammel Neurorehabilitation Centre and University Research Clinic, Hammel, Denmark; Rehabilitation Campus Sint-Ursula, Jessa Hospitals, Herk-de-Stad, Belgium; KU Leuven, University of Leuven, Department of Rehabilitation Sciences, Leuven, Belgium

**Keywords:** Virtual reality, Stroke, Upper extremity, Rehabilitation, Motor function, Physical therapy, Occupational therapy

## Abstract

**Background:**

Novel virtual reality rehabilitation systems provide the potential to increase intensity and offer challenging and motivating tasks. The efficacy of virtual reality systems to improve arm motor function early after stroke has not been demonstrated yet in sufficiently powered studies. The objective of the study is to investigate whether VR training as an adjunct to conventional therapy is more effective in improving arm motor function in the subacute phase after stroke than dose-matched conventional training, to assess patient and therapist satisfaction when working with novel virtual reality training and to calculate cost-effectiveness in terms of resources required to regain some degree of dexterity.

**Methods/Design:**

Randomized controlled observer-blind trial.

One hundred and twenty patients up to 12 weeks after stroke will be randomized to either a group receiving VR training or dose-matched and therapist attention-matched conventional arm training in addition to standard rehabilitation. During a period of four weeks the patients will be offered additional 4–5 training sessions a week of 45–60 minutes duration by a physiotherapist or an occupational therapist.

Study outcomes: Arm motor function, dexterity and independence in daily life activities will be evaluated at baseline, post treatment and three months follow-up assessments with the Action Research Arm Test, Box and Blocks Test and the Functional Independence Measure, respectively. Patient and therapist satisfaction with the implementation of a VR rehabilitation system will also be assessed with questionnaires and interviews.

**Discussion:**

Virtual reality systems are promising tools for rehabilitation of arm motor function after stroke. Their introduction in combination with traditional physical and occupational therapy may enhance recovery after stroke, and at the same time demand little personnel resources to increase training intensity. The VIRTUES trial will provide further evidence of VR-based treatment strategies to clinicians, patients and health economists.

**Trial registration:**

ClinicalTrials.gov NCT02079103

## Background

Early after stroke, approximately two thirds of all patients experience impaired motor function of an upper extremity [1,2]. Only 50% of cognitively intact and medically stable patients with initially reduced upper extremity motor function obtain satisfactory dexterity in the course of the first three months [1,2]. High intensity and large doses of task-related training with many repetitions have been identified as key factors for motor therapy after stroke [3]. Virtual reality (VR) therapy allows therapists to supervise several patients at the same time while providing more intensive training as compared to self-training. VR training has the potential to increase intensity, the range of possible training tasks and may also boost the motivation of patients by adding a playful element to therapy [4]. Learning is facilitated by providing an enriched environment, multisensory stimulation and direct visual or auditory feedback, which seems to be delivered by VR therapy [5]. Furthermore many systems allow for a graded adaption to increasing motor abilities, thus maintaining the challenging character of the tasks.

VR in rehabilitation is a relatively novel approach, and its effectiveness is still under evaluation. Several small scale and proof of principle studies have been performed supporting the application of VR methods in upper limb rehabilitation [6-9]. The largest study to date, including an impressive number of 376 patients, supported the beneficial effect of VR rehabilitation [10]. However, the results have to be interpreted with caution since the patients were not randomized. Reviews by Laver et al. [11], Saposnik et al. [12] and, most recently, Fluet and Deutsch [4] found evidence for improvement of arm motor function and activities of daily living when comparing VR training to various control interventions. However, clinical evidence based on large-scale randomized controlled studies is lacking, especially for patients in the early phase after stroke, when maximal plasticity can be expected [13].

The objectives of this study are:

1. to investigate whether VR training as an adjunct to conventional therapy is more effective in improving arm motor function in the subacute phase after onset of stroke than dose- and therapist attention-matched conventional training;
2. to assess patient and therapist satisfaction when working with novel virtual reality training;
3. to calculate cost-effectiveness in terms of resources required to regain some degree of dexterity.

## Methods/Design

### Design

This study is an international randomized controlled multicenter trial with five participating rehabilitation centers in Denmark, Norway and Belgium. VR training will be compared to conventional upper limb rehabilitation. The VR and the control group will receive the same amount of therapist contact and training time as well as standard and patient-focused inpatient rehabilitation.

### Patient population

All patients with stroke confirmed by clinical neuroimaging admitted for rehabilitation at the participating centers within 1 to 12 weeks after stroke will be considered for inclusion. They will be offered participation in the study if they fulfil the following eligibility criteria:

- First ever ischemic or hemorrhagic stroke or former stroke without any residual motor impairment
- 1 to 12 weeks post stroke
- Impaired arm motor function but some residual arm motor activity as defined by a score of less than 52 on Action Research Arm Test (ARAT), and ability to execute at least 20 degrees of active shoulder flexion and abduction against gravity
- Age 18 years or older
- Able to give informed consent
- No severe cognitive impairment defined as < 20 on Mini Mental State Examination
- No orthopedic impairment, limiting mobility substantially or causing pain in the affected arm
- No visual disorders limiting the ability to comply with treatment regimen

### Randomization

An independent centralized randomization database will provide allocation concealed to the involved clinicians and assessors. A stratified block randomization of severity of paresis will be performed within each center (two groups; mild to moderate paresis = able to extend the wrist at least 20 degrees and the fingers at least 10 degrees from drop hand position, and severe paresis = not able to extend hand and fingers 20 and 10 degrees, respectively).

### Interventions

The additional intervention will be delivered for 4 weeks with 4 to 5 training sessions/week of up to 60 minutes duration including preparation. To allow for some flexibility, 16–20 therapy sessions should be achieved within a maximum of 30 days. In each center designated research therapists will provide the additional training. Patients in both groups will receive standard and patient-focused inpatient rehabilitation besides the additional intervention. The amount of standard rehabilitation, such as physical and occupational therapy and group training actually received, will be registered for each patient.

### The VR system

**Figure 1:**
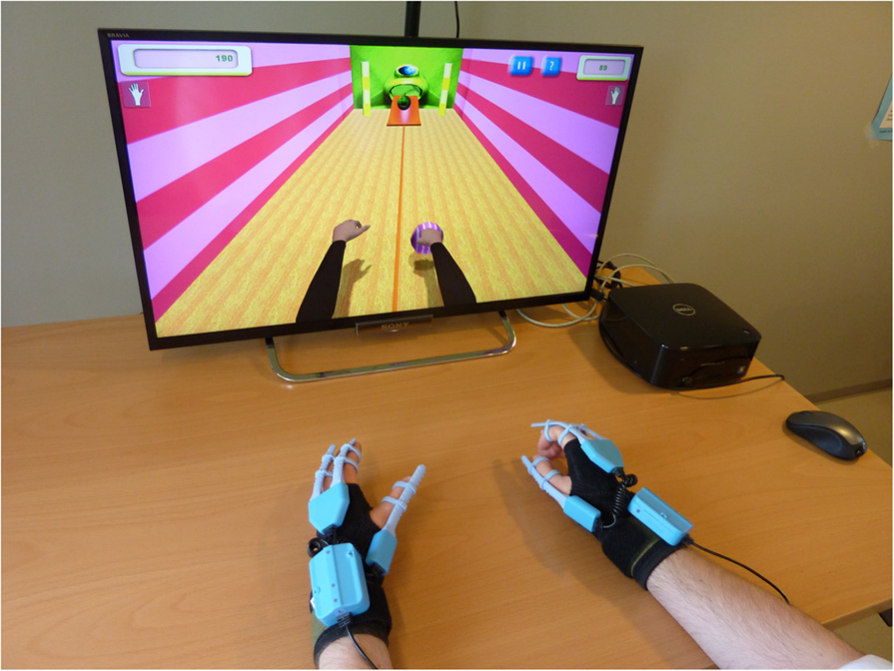
**The virtual reality training system.**

The YouGrabber system (YouRehab Ltd., Switzerland) comprises wearable data gloves with sensors and training software with different gaming alternatives. The system offers several VR rehabilitation scenarios, providing a graded training program of goal-oriented reaching and/or grasping exercises (Figure 1). The YouGrabber was chosen since the different therapy modes provided by the system allow the inclusion of patients with a broad range of arm motor impairments from mildly to severely reduced function. Practical aspects, such as the market and support ability, user friendliness and moderate costs compared to other technology-based rehabilitation products guided the choice for this rehabilitation system.

### Experimental group

Patients randomized to the experimental group participate in VR-based training. The therapist chooses different therapy modes according to the patient’s needs and abilities. Standardized therapy modes comprise unimanual and bimanual training, mirror training and virtually enhanced movements which are performed using different gaming alternatives. The system allows adjusting parameters such as object speed, interval between successive objects, lateral left/right dispersion of object start positions and the probability distribution of the object start positions. During therapy patients are seated at a table facing a monitor, with the arms on the table in front of them.

### Control group

Patients in the control group receive individually tailored conventional training consisting of a self-training program under supervision of a therapist to match the therapy and intensity provided in the experimental group. Conventional arm training is based on a set of standardized exercises which comprise task-related practice for gross movements and dexterity including different grips and selective finger movements, and training in daily life activities. Patients in the control group are also training while seated at a table.

### Outcome measures

All assessments will be performed by assessors blinded to group allocation at baseline (<72 h before intervention start), post treatment (within 72 h), and at three months follow-up (±1 week). Assessors are trained in testing procedures and a comprehensive assessment manual has been developed to ensure standardized performance. Efforts will be made to include all randomized patients at all assessments including those who discontinue the intervention.

The primary outcome measure is the Action Research Arm Test (ARAT), reflecting arm motor function on a broad range of arm and hand activities [14].

Secondary outcome measures include the Box and Blocks Test which is a timed test of dexterity [15]. Independence in activities of daily living will be assessed by the Functional Independence Measure (FIM) [16]. Perceived difficulty performing daily bimanual tasks is assessed with the ABILHAND questionnaire. Patient and therapist satisfaction with the VR training will be assessed by a standardized questionnaire.

**Figure 2:**
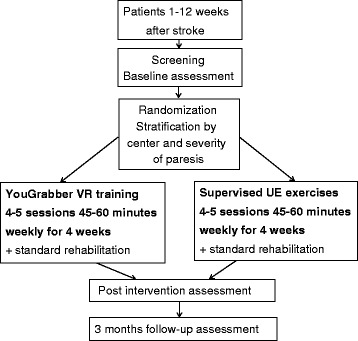
**Flow of patients through the study.**

The study flow chart is presented in Figure 2.

### Data monitoring body

The VIRTUES trial has been approved by the Norwegian Regional Committees for Medical and Health Research (protocol number 2013/0663) and will be conducted in conformance with the “Declaration of Helsinki”. Written informed consent for participation will be obtained. The trial is registered at ClinicalTrials.gov (NCT02079103). An independent steering committee will overview the trial.

### Sample size

The number of participants is based on a clinically meaningful change of 6 points [17] on the ARAT, with a statistical power of 80% and α = 5%. An 8 points SD as an expression of the population variance has been reported for patients early after stroke [18]. In the current study also patients in a later phase post stroke will be included; therefore a larger SD of 11 points was estimated. Power calculations demonstrated a required sample size of 106 patients for two groups; 53 in each group. The recruitment of 120 patients is intended to compensate for 10% drop-outs at three months and the ability to conduct a post-hoc analysis based on the stratification process used according to severity.

### Statistical analysis

Statistical methods to assess differences between groups and within groups will be ANOVA and ANCOVA analyses, t-tests and Chi-square tests. Intention to treat (ITT) and per-protocol analyses will be used. Subgroup analyses according to the stratification “severity” will be performed. Potential confounders, such as time after stroke, treatment time and number of treatment sessions will be included in an ANCOVA. Treatment costs for both groups will be calculated and compared in a cost-effectiveness analysis.

### Study organization

The study is organized and coordinated by the University of Bergen, Norway. Collaborating institutions are Haukeland University Hospital and Sunnaas Rehabilitation Hospital in Norway, Hammel Neurorehabilitation Center and University Research Clinic and Skive Neurorehabilitation in Denmark and the KU Leuven- University of Leuven and Jessa Hospitals in Belgium.

## Discussion

VR training to improve arm motor function after stroke seems to be a promising approach. It provides the opportunity to engage in motivating training with many repetitions, salient stimuli and challenging tasks. These features are supposed to induce adaptive plastic changes [19]. However, there is a dearth of randomized controlled trials documenting its effectiveness [11,12]. VIRTUES is an international multicenter RCT assessing the effectiveness and cost-effectiveness of virtual reality upper limb motor training for patients in the subacute phase after stroke. Key features of the VR system used in this study, such as the playful character, the intensity and the feedback provided are similar to other systems. Thus, the results are expected to be transferable to other systems employing VR rehabilitation for stroke patients.

Most treatment studies on recovery of arm motor function after stroke are limited to patients with mild to moderate impairment [20]. We choose to include patients with more severely impaired motor function, too, since there are few treatment alternatives for this group. The VR system comprises the possibility to virtually enhance movements and to mirror movements of the non-affected arm. The activation of action observation and action execution networks may facilitate motor function as demonstrated in studies applying mirror therapy and action observation [21-23].

Cognitive impairments after stroke are common, either as comorbidity or as a result of the incident. A prevalence of cognitive impairment has been reported in about 50% of all patients in the acute phase after stroke [24]. We set a relatively low cut off of 20 points on MMSE to be able to include a wider range of patients, including those with mild to moderate cognitive impairment, thus trying to enhance the external validity of the study.

The relatively large number of patients included and the multicenter character of the study will increase the generalizability of study results across different rehabilitation centers in different countries. The study will also provide an opportunity to evaluate the feasibility and implementation of VR-based rehabilitation from therapists’ and patients’ points of view. The results of this study will assist in clinical decision making and future practice. Patients and therapists will benefit from improved knowledge about novel treatment strategies.

## Abbreviations

ANCOVA: Analysis of co-variance
ANOVA: Analysis of variance
ARAT: Action research arm test
SD: Standard deviation
VR: Virtual reality
